# Health literacy disparities in South Korea: insights from a latent profile analysis

**DOI:** 10.1186/s12889-025-23025-3

**Published:** 2025-05-17

**Authors:** Sunghyun Kim, Suwon Hwang, Junhyup Lee, Mankyu Choi

**Affiliations:** Department of Health Policy & Management, College of Public Health Science and Transdisciplinary Major in Learning Health Systems, Graduate School, Korea University, Seoul, South Korea

**Keywords:** Health literacy, Latent profile analysis, Chronic disease, Health disparities, Public health

## Abstract

**Background:**

Health literacy has been empirically linked to overall health outcomes. Existing interventions generally employ a variable-centred approach, often neglecting the cumulative impact of various factors and the ways different groups interact with health information. We aimed to explore health literacy in the general population of South Korea by identifying and characterising distinct health literacy profiles using latent profile analysis.

**Methods:**

A cross-sectional design was utilised, analysing health literacy data from the second wave of the Korea Health Panel Survey (collected between March and July 2021) with responses from 9,509 adults. Health literacy was assessed in the domains of health management, disease prevention, and health promotion using the 16-item European Health Literacy Survey Questionnaire. Latent profile analysis was used to identify health literacy profiles, and multinomial logistic regression analysis was used to examine sociodemographic and health-related factors associated with profile membership.

**Results:**

Latent profile analysis revealed three distinct health literacy groups: low (27.91%), moderate (63.51%), and high (8.58%). A social gradient was observed, with disparities in age, education, income, and residence observed across the groups. Membership in the low health literacy group was associated with being older, disability, and chronic disease—populations with heightened healthcare needs.

**Conclusions:**

The findings underscore the need for targeted interventions to address the unique challenges faced by populations with low health literacy in a universal health coverage system such as that of South Korea. By identifying those at highest risk for low HLit and targeting policy measures accordingly, healthcare systems can allocate resources more effectively and make health information genuinely accessible for all. In doing so, such strategies can ultimately help mitigate the disparities highlighted in this study. These insights provide a foundation for policies aimed at promoting health equity through focused health literacy initiatives.

## Background

Health literacy (HLit)—the ability to obtain, understand, and apply health information for informed decision-making in the domains of health management, disease prevention, and health promotion [1]—has been empirically linked to overall health outcomes. Poor HLit in individuals is associated with adverse health outcomes, increased healthcare costs, and higher mortality rates [2–6]. HLit has also been recognised as important in addressing broader population-level problems, with the World Health Organization Commission on Social Determinants of Health highlighting it as central to addressing global health inequities [7]. However, despite ongoing attention, nearly half of the adult population in the US, Europe, and Asia still experiences inadequate levels of HLit [8–13].

To date, policies on HLit have provided knowledge, information, and skills—mainly in clinical settings—or have improved HLit in various populations [14–16]. These interventions generally employ a variable-centred approach, focusing on demographic factors identified as key predictors of HLit, such as socioeconomic status, education, and age [10]. While foundational, such methods are limited in addressing the complex, heterogeneous nature of HLit, often neglecting the cumulative impact of various factors and the ways different groups interact with health information [17, 18]. Despite HLit having been identified as playing a mediating role in health disparities, the abovementioned gap has limited our ability to appropriately address HLit at the population level to ensure equitable health outcomes [19, 20].

Despite South Korea’s increasing universal healthcare coverage to improve healthcare access, low levels of HLit continue to be a challenge for equitable health outcomes as in other countries [21–24]. One aspect of the problem is an inadequate understanding of varying HLit levels across the population, stemming from limited evidence on the disparities present and approaches to address issues [22, 25]. Although South Korea’s recent large-scale HLit survey represents a critical step forward, there is still much to be explored in terms of disparities in the population and specific subgroups [24, 25]. Consequently, approaches that can accurately characterise HLit at the population level and account for diverse HLit groups are essential to pave the way for targeted, contextually relevant support that goes beyond demographic predictors alone [26].

Recent literature on HLit highlights the need for a comprehensive, person-centred approach that considers the interplay of individual characteristics and sociodemographic and health-related factors [17, 26–28]. However, previous studies have relied on total or average HLit scores measured under various criteria, which may lead to overlooking important subgroup differences [9, 10, 17]. This emphasises the value of clustering methods, such as latent profile analysis (LPA), which enable the person-centred categorisation of individuals into subgroups based on shared, often unseen attributes. LPA also offers a statistically objective method to identify subgroups or classes of individuals, thereby capturing the underlying heterogeneity of the population by identifying relatively homogeneous subgroups and its proportions [27, 29]. Unlike traditional methods, LPA can uncover diverse profiles within a population, providing more insights into the disparities present [29].

However, despite its potential, LPA remains underutilised in HLit research. Studies have typically focused on specific diseases or demographic groups, and to our knowledge, there has been no attempt yet at utilising LPA in a population-level analysis of HLit and a significant gap remains in this area [27, 28, 30, 31]. Expanding its use could provide valuable insights into more scalable and effective interventions that can address the diverse HLit needs at the population level [32]. This is particularly relevant in South Korea, where diverse population needs in a universal health coverage system remain underserved by one-size-fits-all approaches. Consequently, subgroup-specific insights such as LPA could allow us to address diverse HLit needs more precisely and equitably.

Against this background, we employ LPA as an approach to identify distinct HLit profiles and analyse the demographic and health-related factors that characterise each. Through this, the present study aims to advance the literature on HLit by offering a better understanding of distinct profiles and characteristics, which was otherwise limited in previous studies—especially at the broad population level. We anticipate that these findings will serve as a foundation for more targeted HLit policies and interventions.

## Methods

### Aim

The following questions are addressed: (1) What distinct HLIT profiles can be identified in the general population using LPA, and how do these profiles differ in sociodemographic and health-related characteristics? (2) What factors are associated with membership in these different HLit profiles? By answering these questions, we aim to provide insights into disparate HLit groups within the population, which could be a foundation for creating more targeted, effective HLit interventions.

### Participants and data collection

We utilised data from the Korea Health Panel Survey (KHPS), a nationally representative survey that examines factors influencing healthcare use, including socioeconomic characteristics, comorbidities, and health behaviours [33]. Using the 16-item version of the European Health Literacy Survey Questionnaire (HLS-EU-Q16), the KHPS has been measuring HLit in South Koreans aged 19 and above since 2020 [24]. We used the most recent HLit data collected between March and July 2021 using computer-assisted personal interviews by trained interviewers. Participants in the KHPS were selected using the 2016 registered census as the sampling frame to reflect the changed population structure [33]. The samples were extracted from 708 survey districts using a two-stage stratified cluster sampling design. Stratification was performed twice—first, based on the large administrative district (city/province) and then, based on a smaller district. From the 11,057 responses recorded, those with missing response across any of the 16 questions of the HLit questionnaire were excluded in our analysis. Thus, the final sample size was 9,509 participants.

### HLit

HLit was assessed using the HLS-EU-Q16, originally developed to measure HLit in the European population, which has been increasingly used and verified in South Korea [24, 34, 35]. The instrument comprehensively measures HLit across three domains: health management, disease prevention, and health promotion as proposed by Sørensen’s integrated model of HLit which emphasize individuals’ capacity to access, understand, appraise, and apply health information for different health-related tasks [1]. This multidimensional structure has been widely applied and has been used to create the HLS-EU-Q16 subsequently. The HLS-EU-Q16 is particularly suited for population-based research owing to its short administration time [13, 32]. The instrument consists of 16 items, each rated on a 4-point Likert scale (1 = very difficult to 4 = very easy) with an additional “I don’t know” option, asking participants how easily they can access, understand, appraise, and apply health-related information. Following previous research indicating that “I don’t know” responses are likely non-substantive rather than reflective of low HLit, we treated them as missing data and excluded any respondents who selected “I don’t know” from our analysis [24, 35]. For our analysis, we calculated individual mean scores (range 1–4) for each of the three HLit domains, with higher scores indicating greater HLit levels. The HLS-EU-Q16 was translated into Korean using a forward–backward translation method, yielding high internal consistency (Cronbach’s alpha = 0.861) [34].

### Sociodemographic and health-related characteristics

Based on theoretical and empirical evidence [7, 9, 10, 12, 19, 23], the following variables were included: sex, age, marital status, residential area, educational level, employment status, annual household income, health coverage type, and presence of disability and chronic disease. These variables can either enhance or hinder HLit and have also been recognized in previous studies as key predictors [9, 10, 19]. Older age and lower socioeconomic status have consistently been linked to lower levels of HLit by limiting access to and use of health-related information, while differences across various sociodemographic factors have also been documented [7, 12, 15]. Moreover, health coverage status, disability, and chronic disease can influence HLit by introducing distinct challenges or needs, underscoring their importance in determining HLit levels [4, 6, 9]. Age was categorised into two groups: below 65 years and 65 years or older. Marital status was classified as either married or single (including separated, widowed, divorced, and never married). Residential area was classified as urban and rural. Educational level was dichotomised into high school or below and university or above. Employment status was grouped into employed and not employed. Health coverage type was classified as either receiving medical aid (a form of public medical assistance for the underprivileged population in South Korea) or having mandatory national health insurance. The presence of disability and chronic disease was also included. Annual household income was log-transformed to address skewness and improve distribution normality using the natural logarithm. Specific measurement items are detailed in the *KHPS (Second Wave) User Guide* [33].

### Statistical analyses

LPA was employed as a person-centred approach to categorise individuals into distinct profiles based on the three dimensions of HLit as indicator variables. While previous studies have often relied on conventional cutoff points or simple subgroup definitions of HLit (e.g., inadequate vs. adequate), these variable-centred approaches may fail to capture the complexity of how different HLit domains cluster within individuals [12, 17, 26–28]. Unlike traditional methods focused on variable relationships, LPA identifies subgroups within a heterogeneous population where members share similar latent attributes—those underlying characteristics that are not directly observable. Because LPA uses continuous indicators and accounts for measurement error, it provides finer distinctions in group classification, uncovering nuanced subgroups. LPA facilitates the examination of meaningful differences in characteristics and outcomes across these subgroups.

Modelling began with a single profile and was progressively increased to six profiles to determine the optimal model fit. Model fit was assessed using the Akaike information criterion (AIC), Bayesian information criterion (BIC), sample size-adjusted BIC (SSABIC), and entropy. Lower values of AIC, BIC, and SSABIC indicate improved model fit, and an entropy value above 0.76 signifies high classification accuracy. To compare profile models, the Lo–Mendell–Rubin adjusted likelihood ratio test (LMRT) and the bootstrap likelihood ratio test (BLRT) were used, with a p-value below 0.05 indicating that a model with k profiles fits better than one with k-1 profiles. LPA was performed using the ‘tidyLPA’ package in R.

To examine differences in characteristics among the identified HLit profiles, the Rao–Scott chi-square test and survey-adjusted analysis of variance were employed to handle the complex survey data. Additionally, mean scores and standard deviations for HLit dimensions and individual questions were analysed across the profiles. Multinomial logistic regression analysis was conducted to identify factors associated with profile membership, with adjusted odds ratios (ORs) and 95% confidence intervals (CIs) calculated. Variables were selected based on theoretical relevance, and no stepwise procedures were used. Assumptions for each analysis were met. Normality was assessed using the Shapiro–Wilk test, homogeneity of variances using Levene’s test, and multicollinearity using variance inflation factors. All statistical tests were two-tailed, with the significance level set at *p* < 0.05.

All statistical analyses were performed using R Version 4.4.2 and STATA 18 (StataCorp., College Station, TX, USA). Survey weights, representing the inverse of individual selection probability, were incorporated as provided in the 2021 KHPS dataset. The Institutional Review Board of Korea University approved the study protocol, granting an IRB exemption and waiving the requirement for informed consent (reference No. KU_IRB-2024-0223), owing to the use of publicly available secondary data from the KHPS. The KHPS obtained informed written consent from participants during the original data collection process, which covered the use of survey data for research purposes. The dataset was anonymised by the KHPS prior to being made accessible to researchers, and no direct interaction with participants occurred during this study.

## Results

### LPA and HLit groups

For the LPA, models with one to six profiles were tested incrementally (Table 1). As the number of profiles increased, AIC, BIC, and SSABIC values gradually decreased, indicating improved model fit. The three-profile model was selected as the optimal fit, based on the highest entropy value (0.918) and because each profile accounted for more than 5% of the sample, demonstrating strong classification accuracy and clear profile separation. The decision was further supported by significant LMRT and BLRT values (*p* < 0.01), underscoring the model’s robustness.

**Table 1:** Model fit indices for the latent profile analysis of health literacy

Number of profiles	LL	df	AIC	BIC	SSABIC	Entropy	LMRT	BLRT
1	-22,057.519	2	44,127.039	44,169.999	44,150.932	1.000	-	-
2	-17,203.982	5	34,427.963	34,499.563	34,467.785	0.808	< 0.001	< 0.0099
3	-12,510.624	8	25,049.248	25,149.488	25,104.998	0.918	< 0.001	< 0.0099
4	-10,740.276	11	21,516.551	21,645.431	21,588.230	0.893	< 0.001	< 0.0099
5	-9,566.504	14	19,177.008	19,334.528	19,264.615	0.891	< 0.001	< 0.0099
6	-8,994.366	17	18,040.732	18,226.892	18,144.268	0.896	< 0.001	< 0.0099

Note: LL, log likelihood; df, degree of freedom; AIC, Akaike information criterion; BIC, Bayesian information criterion; SSABIC, sample size-adjusted BIC; LMRT, Lo–Mendell–Rubin likelihood ratio test; BLRT, bootstrap likelihood ratio test

**Fig. 1:**
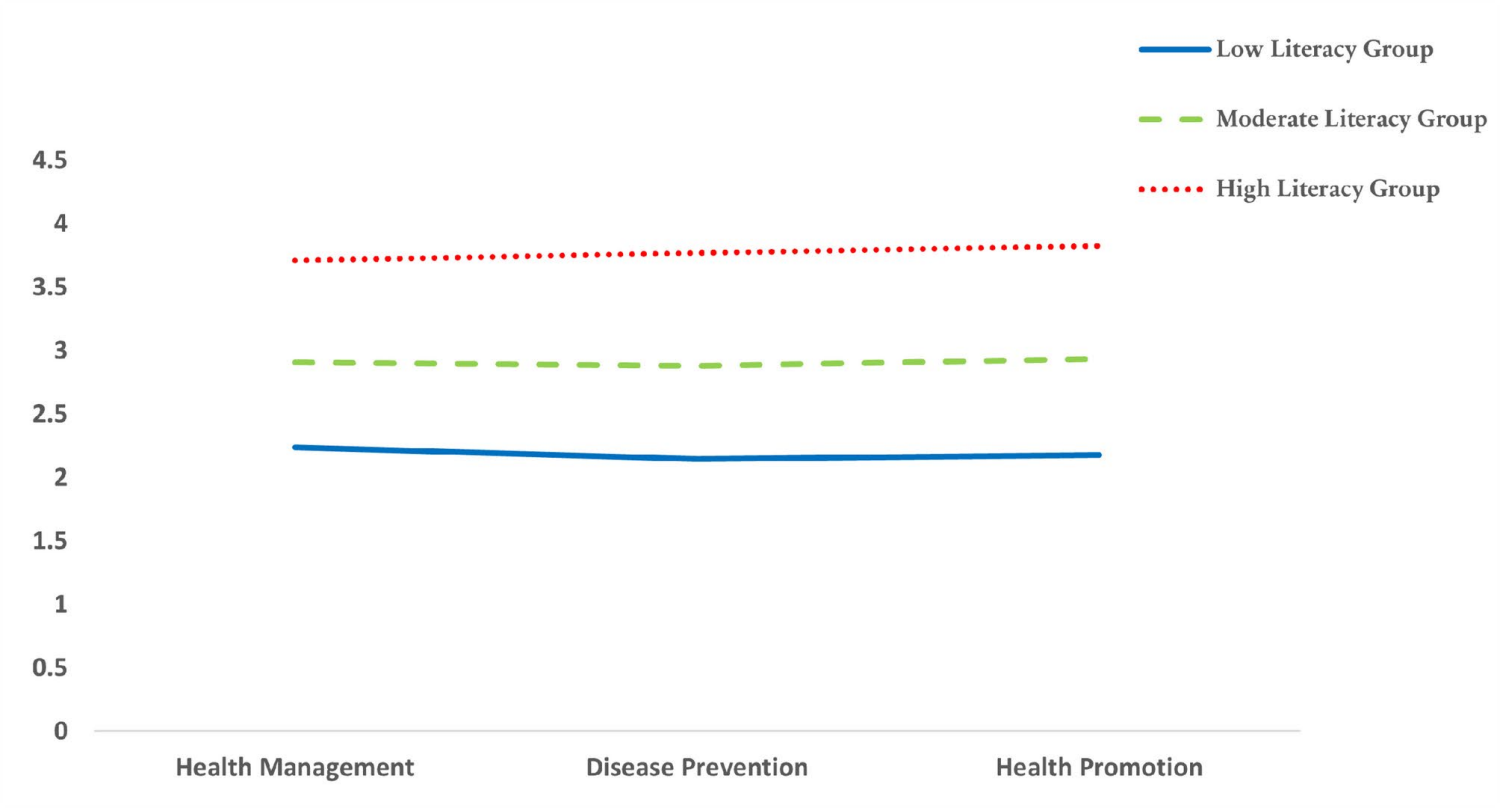
Three-profile model of health literacy (*n* = 9,509)

The results of the three-profile model of HLit are shown in Fig. 1. The first profile, comprising 27.91% of the sample (*n* = 2,654), was characterised by lower scores across all dimensions and was thus labelled the ‘low HLit’ group. The second profile, comprising 63.51% of the sample (*n* = 6,039), showed moderate scores across all dimensions and was labelled the ‘moderate HLit’ group. The third profile, representing 8.58% of the sample (*n* = 816), had higher scores across all dimensions and was labelled the ‘high HLit’ group. The sum scores of the mean (Table 2) across all dimensions were 6.56 for the low HLit group, a large gap compared with the moderate (sum score: 8.72) and high (sum score: 11.30) HLit groups. In terms of HLit dimensions, the low and moderate HLIT groups found disease prevention the most difficult, while the high HLit group found health management challenging.

**Table 2:** Mean score of the health literacy items for each group

Health literacy items		Low health literacy (*n* = 2,654)	Moderate health literacy (*n* = 6,039)	High health literacy (*n* = 816)
		M (SD)	M (SD)	M (SD)
**Total mean health literacy score across dimensions**		6.56 (0.84)	8.72 (0.59)	11.30 (0.64)
**Mean health literacy score for health management**		2.24 (0.35)	2.90 (0.28)	3.71 (0.32)
	Find information on treatments of illnesses that concern you.	1.82 (0.58)	2.77 (0.55)	3.58 (0.55)
	Find out where to get professional help when you are ill.	1.98 (0.63)	2.80 (0.52)	3.64 (0.52)
	Understand what your doctor says to you.	2.55 (0.59)	3.04 (0.41)	3.77 (0.44)
	Understand your doctor or pharmacist’s instructions on how to take a prescribed medicine.	2.73 (0.56)	3.14 (0.42)	3.86 (0.38)
	Judge when you may need to get a second opinion from another doctor.	1.88 (0.54)	2.67 (0.54)	3.56 (0.58)
	Use information the doctor gives you to make decisions about your illness.	1.96 (0.52)	2.80 (0.47)	3.68 (0.49)
	Follow instructions from your doctor or pharmacist.	2.73 (0.55)	3.11 (0.44)	3.88 (0.36)
**Mean health literacy score for disease prevention**		2.15 (0.34)	2.88 (0.27)	3.77 (0.26)
	Find information on how to manage mental health problems such as stress or depression.	1.87 (0.49)	2.75 (0.51)	3.73 (0.48)
	Understand health warnings about behaviour such as smoking, low physical activity, and drinking too much.	2.29 (0.61)	3.03 (0.41)	3.88 (0.34)
	Understand why you need health screenings.	2.66 (0.59)	3.15 (0.41)	3.93 (0.26)
	Judge if the information on health risks in the media is reliable.	1.99 (0.52)	2.72 (0.50)	3.65 (0.52)
	Decide how you can protect yourself from illness based on information in the media.	1.92 (0.44)	2.73 (0.48)	3.66 (0.50)
**Mean health literacy score for health promotion**		2.18 (0.34)	2.94 (0.22)	3.82 (0.25)
	Find out about activities that are good for your mental well-being.	1.95 (0.49)	2.86 (0.42)	3.78 (0.42)
	Understand advice on health from family members or friends.	2.61 (0.56)	3.07 (0.34)	3.88 (0.33)
	Understand information in the media on how to get healthier.	2.10 (0.54)	2.94 (0.35)	3.84 (0.37)
	Judge which everyday behaviour is related to your health.	2.05 (0.50)	2.88 (0.41)	3.78 (0.42)

Note: M, mean; SD, standard deviation

### Distribution of sociodemographic and health-related characteristics across HLit groups

Table 3 presents the results of the comparison of characteristics among the three latent profiles, with significant differences across all variables. Distinct variations were particularly noted in the low HLit group, with a higher prevalence of female, older adults, non-economically active individuals, medical aid receivers, and higher rates of disability and chronic disease. Conversely, this group exhibited lower proportions of higher education, income, and urban residency.

**Table 3:** Comparison of sociodemographic and health-related characteristics among the health literacy groups

Characteristics		Profile 1		Profile 2		Profile 3		*p*-value
		Low health literacy (*n* = 2,654)		Moderate health literacy (*n* = 6,039)		High health literacy (*n* = 816)		
		*n*	% (Weighted)	*n*	% (Weighted)	*n*	% (Weighted)	
**Sex**	**Male**	974	37.34	2,851	48.01	422	51.05	< 0.001
	**Female**	1,680	62.66	3,188	51.99	394	48.95	
**Age (years)**	**< 65**	666	44.19	4,277	85.50	757	96.40	< 0.001
	**≥ 65**	1,988	55.81	1,762	14.50	59	3.60	
**Marital status**	**Single, divorced, separated,** **or widowed**	912	42.43	1,684	34.27	303	39.89	< 0.001
	**Married**	1,742	57.57	4,355	65.73	513	60.11	
**Region**	**Rural**	1,678	58.05	3,214	54.34	375	49.18	0.002
	**Urban**	976	41.95	2,825	45.66	441	50.82	
**Educational** **level**	**High school or below**	2,408	82.48	3,540	46.42	215	23.55	< 0.001
	**University or above**	246	17.52	2,499	53.58	601	76.45	
**Economic** **activity**	**No**	1,484	54.18	2,232	32.14	234	28.65	< 0.001
	**Yes**	1,170	45.82	3,807	67.86	582	71.35	
**Log (Income)** ^*^		7.84 ± 0.89		8.45 ± 0.80		8.75 ± 0.73		< 0.001
**Type of** **health coverage**	**Medical aid**	205	8.57	182	2.39	13	1.15	< 0.001
	**National health insurance**	2,449	91.43	5,857	97.61	803	98.85	
**Disability**	**No**	2,290	87.91	5,735	96.58	805	99.34	< 0.001
	**Yes**	364	12.09	304	3.42	11	0.66	
**Chronic disease**	**No**	609	32.50	3,393	67.82	666	83.87	< 0.001
	**Yes**	2,045	67.50	2,646	32.18	150	16.13	

Note: p-values were estimated using the Rao–Scott chi-square test and survey-adjusted analysis of variance

* Income was log-transformed and presented as mean ± standard deviation

### Factors associated with HLit group membership

Logistic regression was conducted to analyse factors associated with group membership (Table 4). The results showed that, compared with the low HLit group, females and older individuals (aged 65 and above) had significantly lower odds of belonging to the moderate and high HLit groups (*p* < 0.01). A similar trend was observed for higher educational and income levels, associated with an increased likelihood of being in the moderate and high HLit groups (*p* < 0.001). Additionally, the presence of disability and chronic disease significantly decreased the odds of belonging to the moderate and high HLit groups (*p* < 0.001). Urban residence, economic activity, or having national health insurance was associated with an increased likelihood of being in the moderate HLit group only (*p* < 0.05). Marital status was not statistically significant in our regression model.

**Table 4:** Results of multinomial logistic regression analysis

Characteristics		Low vs. moderate health literacy				Low vs. high health literacy			
		OR	95% CI		*p*-value	OR	95% CI		*p*-value
**Sex**	**Male**	Reference							
	**Female**	0.768	0.658	0.897	0.001	0.702	0.551	0.894	0.004
**Age (years)**	**< 65**	Reference							
	**≥ 65**	0.315	0.269	0.369	< 0.001	0.152	0.102	0.226	< 0.001
**Marital status**	**Single, divorced, separated,** **or widowed**	Reference							
	**Married**	1.137	0.967	1.338	0.121	0.892	0.695	1.144	0.367
**Region**	**Rural**	Reference							
	**Urban**	1.137	0.995	1.342	0.058	1.306	1.038	1.642	0.023
**Educational** **level**	**High school or below**	Reference							
	**University or above**	2.339	1.909	2.865	< 0.001	4.376	3.288	5.824	< 0.001
**Economic** **activity**	**No**	Reference							
	**Yes**	1.258	1.075	1.472	0.004	1.179	0.908	1.531	0.215
**Log (Income)**		1.284	1.170	1.410	< 0.001	1.786	1.502	2.125	< 0.001
**Type of** **health coverage**	**Medical aid**	Reference							
	**National health insurance**	1.448	1.076	1.949	0.015	1.321	0.641	2.723	0.450
**Disability**	**No**	Reference							
	**Yes**	0.592	0.464	0.754	< 0.001	0.200	0.091	0.437	< 0.001
**Chronic disease**	**No**	Reference							
	**Yes**	0.596	0.505	0.704	< 0.001	0.394	0.295	0.526	< 0.001

Note: OR, odds ratio; CI, confidence interval

## Discussion

We employed LPA to identify distinct HLit profiles in the general population in South Korea. The three-profile model was selected as the optimal fit, with distinct low (27.91%), moderate (63.51%), and high (8.58%) HLit groups, providing insights into different HLit groups in universal health coverage systems such as in South Korea, where HLit patterns are often generalised [22, 24]. Our findings align with those of previous studies regarding nationwide HLit distributions [9, 11, 12, 24, 32, 36]. The latent profiles identified underscore the existing disparities in HLit levels and support the need for nuanced, person-centred approaches that consider the cumulative effects of individual characteristics and sociodemographic and health-related factors. Our analysis further reveals distinct differences across these profiles and highlights the factors associated with membership in each HLit group.

First, the low HLit group had a substantially lower overall HLit mean score compared with the moderate and high HLit groups, underscoring an urgent need to prioritise this population. This group also had markedly lower scores across all HLit dimensions, pointing to potential disparities in healthcare access, quality, and overall health outcomes. These findings highlight systemic inequities in HLit that demand focused policy interventions. Previous research shows that individuals with limited HLit face greater difficulties accessing health information, adhering to self-care methods, and navigating the healthcare system [3, 5, 6, 37]. Such disparities often stem from and are exacerbated by systemic issues—including socioeconomic, educational, and health-related factors—that reinforce barriers to effective healthcare utilisation. These disparities may continue to widen without intervention, additionally burdening healthcare systems and deepening health inequities.

Our findings also contribute to the growing body of evidence on sociodemographic and economic factors that influence HLit, identifying a clear social gradient among the HLit groups, consistent with all previous nationwide HLit research globally [12, 19, 24, 36, 38]. Specifically, gender, age, place of residence, educational level, economic activity, and income significantly differed across our HLit groups. Low socioeconomic status and older age are often associated with limited HLit, further challenging individuals’ ability to engage effectively with health services [34, 39]. Such populations face unique barriers, including decreased access to healthcare resources and education, which complicates their ability to interpret and utilise health information effectively. While enhancing HLit alone is not the solution to eliminating the root causes of health disparities, it has been suggested to mediate the relationship between socioeconomic disadvantages and access to healthcare services, inadequate health-related behaviours, and poor health outcomes [20, 40, 41]. Enhancing HLit could, therefore, be an intervenable factor to reduce negative health impacts and inequities tied to socioeconomic disadvantage.

A key finding is that the group with the lowest HLit predominantly includes individuals who demand the most healthcare services, such as older adults and those with disabilities and chronic diseases. According to Nutbeam, priority should be proportionate to need, engaging and supporting groups disproportionately affected by low HLit [20]. Thus, interventions must be targeted at these vulnerable groups to enhance HLit, enabling them to engage more effectively with health information and healthcare systems [34, 42]. The individuals identified in our analysis may face significant challenges in navigating complex health information, medication regimens, self-care instructions, and the broader healthcare system. Their difficulties may be further exacerbated by ageing or multiple chronic diseases associated with a diminished capacity to access and utilise health information [37, 39].

While the South Korean government has strengthened its universal health insurance coverage to improve healthcare access—mainly through reduced out-of-pocket costs especially on vulnerable populations—our findings indicate that low HLit persists, which could be a barrier to these individuals. This suggests that despite efforts, individuals with low HLit may still struggle to access, understand, or navigate healthcare resources fully, hindering efficient resource use [21, 43, 44]. Thus, interventions must go beyond universal accessibility to intensively focus on those at higher risk. A shift in focus from universal to targeted efforts could also aid in reducing inefficient resource utilisation and support the financial sustainability of healthcare systems, reinforcing the goals of universal health coverage.

Despite its contributions, this study has some limitations. First, while the analysis demonstrates that individuals with higher healthcare needs have lower HLit levels, it does not establish a causal link between them owing to the cross-sectional nature of the data. Further research is needed to explore the causal relationships and directionality among HLit and specific healthcare demands, which may benefit policies for expanding health coverage. Longitudinal studies with extended data collection periods could identify these connections, providing insights into optimising universal health insurance coverage strategies to improve effectiveness and resource allocation in South Korea’s healthcare system.

## Conclusions

To our knowledge, this is the first study to employ clustering methods such as LPA in a population-level HLit survey. This study also offers the first comprehensive analysis of HLit across South Korea’s population, identifying distinct HLit groups that highlight significant disparities. Our findings show that individuals with greater healthcare needs—particularly older adults and those with disabilities or chronic diseases—are predominantly in the low HLit group. This stands true despite efforts to increase healthcare access through a universal health coverage system. This underscores the need for efforts targeted at vulnerable groups to reduce HLit gaps and their consequent negative health outcomes in our society. Our findings suggest that policymakers must tailor interventions to the specific needs of lower-HLit groups rather than relying on broad, universal strategies. Doing so could help reduce inefficient resource utilization, improve patient outcomes, and enhance overall equity in healthcare. By systematically identifying at-risk populations, targeted programs—such as simplified health information materials, enhanced patient education, or specialized support services—can be implemented more effectively. In turn, these efforts not only promote better HLit but also have the potential to mitigate social and economic barriers, ultimately contributing to more equitable health outcomes at the population level. Furthermore, these findings may inform HLit policies, especially in countries with similar universal health coverage models, by underscoring the importance of identifying and addressing the unique needs of vulnerable populations. By doing so, we can enhance healthcare access, optimise resources, and support more equitable health outcomes. The insights provided here serve as a foundation for policies aimed at reducing disparities and promoting health equity through focused HLit initiatives.

## Data Availability

The data is owned by a third party and the authors had no special access privileges others would not have. A request for the data used for this study can be made on https://www.khp.re.kr:444/eng/data/data.do. Inquiries regarding data acquisition should be sent to the Korea Institute for Health and Social Affairs (email: khp@kihasa.re.kr).

## Abbreviations

AIC: Akaike information criterion
BIC: Bayesian information criterion
BLRT: Bootstrap likelihood ratio test
HLit: Health literacy
LMRT: Lo–Mendell–Rubin likelihood ratio test
LPA: Latent profile analysis
SSABIC: Sample size-adjusted Bayesian information criterion
